# Cross-Bridges and Sarcomeric Non-cross-bridge Structures Contribute to Increased Work in Stretch-Shortening Cycles

**DOI:** 10.3389/fphys.2020.00921

**Published:** 2020-07-28

**Authors:** André Tomalka, Sven Weidner, Daniel Hahn, Wolfgang Seiberl, Tobias Siebert

**Affiliations:** ^1^Department of Motion and Exercise Science, University of Stuttgart, Stuttgart, Germany; ^2^Human Movement Science, Faculty of Sports Science, Ruhr University Bochum, Bochum, Germany; ^3^School of Human Movement and Nutrition Sciences, University of Queensland, Brisbane, QLD, Australia; ^4^Human Movement Science, Bundeswehr University Munich, Munich, Germany

**Keywords:** contractile behavior, cross-bridge inhibitor, work expenditure, muscle stretch, muscle shortening, history-effects, rFE, rFD

## Abstract

Stretch-shortening cycles (SSCs) refer to the muscle action when an active muscle stretch is immediately followed by active muscle shortening. This combination of eccentric and concentric contractions is the most important type of daily muscle action and plays a significant role in natural locomotion such as walking, running or jumping. SSCs are used in human and animal movements especially when a high movement speed or economy is required. A key feature of SSCs is the increase in muscular force and work during the concentric phase of a SSC by more than 50% compared with concentric muscle actions without prior stretch (SSC-effect). This improved muscle capability is related to various mechanisms, including pre-activation, stretch-reflex responses and elastic recoil from serial elastic tissues. Moreover, it is assumed that a significant contribution to enhanced muscle capability lies in the sarcomeres itself. Thus, we investigated the force output and work produced by single skinned fibers of rat soleus muscles during and after ramp contractions at a constant velocity. Shortening, lengthening, and SSCs were performed under physiological boundary conditions with 85% of the maximum shortening velocity and stretch-shortening magnitudes of 18% of the optimum muscle length. The different contributions of cross-bridge (XB) and non-cross-bridge (non-XB) structures to the total muscle force were identified by using Blebbistatin. The experiments revealed three main results: (*i*) partial detachment of XBs during the eccentric phase of a SSC, (*ii*) significantly enhanced forces and mechanical work during the concentric phase of SSCs compared with shortening contractions with and without XB-inhibition, and (*iii*) no residual force depression after SSCs. The results obtained by administering Blebbistatin propose a titin-actin interaction that depends on XB-binding or active XB-based force production. The findings of this study further suggest that enhanced forces generated during the active lengthening phase of SSCs persist during the subsequent shortening phase, thereby contributing to enhanced work. Accordingly, our data support the hypothesis that sarcomeric mechanisms related to residual force enhancement also contribute to the SSC-effect. The preload of the titin molecule, acting as molecular spring, might be part of that mechanism by increasing the mechanical efficiency of work during physiological SSCs.

## Introduction

Residual force depression (rFD) following active muscle shortening and residual force enhancement (rFE) following active muscle stretch (Abbott and Aubert, 1952) are fundamentally accepted mechanical properties of skeletal muscle (Rassier, 2017). These phenomena have been consistently investigated across all structural levels of muscle from *in vitro* single sarcomeres (Joumaa et al., 2008a) to *in vivo* human multi-joint-contractions (Seiberl et al., 2015a; Chen et al., 2019) [for recent reviews, see Herzog et al., 2016; Rassier, 2017; for further information of the potential mechanisms of r(FD) and r(FE), see Appendix, Supplementary Text S1].

However, stretch-hold (referring to rFE) or shortening-hold (referring to rFD) movements have no real everyday significance. On the contrary, SSCs — eccentric muscle action immediately followed by concentric muscle action — play a significant role in natural locomotion. SSCs represent an essential part of fundamental cyclic movement patterns such as walking, running or jumping (Komi, 2000). Typically, under physiological conditions, a SSC is a rather fast type of contraction during rapid movements (due to short ground contact times of the legs) (Bobbert et al., 1986; van Ingen Schenau et al., 1997; Komi, 2000), while the muscles operating range covers the ascending limb and the plateau-region of their force-length-relation (Burkholder and Lieber, 2001; Kurokawa et al., 2003). An essential feature of the SSC is that the muscular force and work during the concentric phase can be increased by more than 50% compared with concentric muscle actions without preceding stretch (Cavagna et al., 1968; Bosco et al., 1987; Gregor et al., 1988). This SSC-effect (increased muscular capability) is associated with enhanced efficiency accompanied by reduced metabolic energy consumption (Cavagna et al., 1968; Joumaa and Herzog, 2013). However, despite clear evidence concerning increased SSC-effects in various experimental human and animal studies on different structural levels (*in vitro, in situ, in vivo*), the underlying mechanisms remain controversial. This dispute is because none of the currently accepted mechanisms (such as preactivation, stretch-reflex responses, and elastic recoil from serial elastic tissues) can entirely explain the enhanced force response and the increased mechanical work output during SSCs (van Ingen Schenau et al., 1997; Cormie et al., 2011; Seiberl et al., 2015b). Since it was shown early that SSCs provoke increased force/work output in isolated muscle tissue preparations with essentially no tendon, the phenomenon was revisited with approaches designed to identify mechanisms not related to reflex activity or elastic energy recoil, but thought to lie within the sarcomere itself (Cavagna et al., 1968; Seiberl et al., 2015b). This assumption is supported by recent findings, which have shown SSC-effects even on the fiber level (i.e., without serial-elastic components such as the tendon and aponeurosis) (Fukutani and Herzog, 2019).

A potential mechanism to explain the SSC-effect within the sarcomere might be based on different myosin states. Recent X-ray diffraction studies on actively contracting fibers from striated skeletal muscle (Haselgrove, 1975; Linari et al., 2015; Fusi et al., 2016) suggest that the myosin filament can exist in one of two possible states: a relaxed state (OFF) and an activated state (ON). The force response upon muscle stretching, which occurs during the eccentric phase of a SSC, might be affected by the mechanosensitive contributions of XB activation and binding from the myosin OFF into the myosin ON state with stretch (Linari et al., 2015; Fusi et al., 2016; Brunello et al., 2020). Current findings suggest that this regulatory mechanism of thick filament mechano-sensing in striated muscles acts independently of the well-known thin filament-mediated calcium-signaling pathway (Fusi et al., 2016).

Furthermore, there is extensive evidence that the semi-active protein titin (Maruyama et al., 1976) mediates the phenomena of enhanced force response during and following stretch contractions [(r)FE] in skeletal muscle (Tomalka et al., 2017; Herzog, 2018; Freundt and Linke, 2019). Various model approaches (Rode et al., 2009; Nishikawa et al., 2012; Schappacher-Tilp et al., 2015) have been proposed that explain rFE in skeletal muscle and these model approaches are supported by experimental evidence for titin-actin interactions upon muscle activation (Nagy, 2004; Bianco et al., 2007; Dutta et al., 2018; Li et al., 2018; Tahir et al., 2020). Recent studies (Fukutani et al., 2017; Fukutani and Herzog, 2019) suggested that increased rFE is positively related to an increase in force/work during SSCs. These results come from *in vitro* muscle fiber experiments under limited conditions (very slow contraction velocities while the fibers operate mostly on the descending limb of the force-length-relation). There also exist contradictory *in situ* investigations on cat soleus (slow contraction velocities at the ascending limb of the force-length-relation), showing that the effect of rFE disappears as soon as the muscle actively shortens during SSCs (Lee et al., 2001). Hence, it is still controversial whether and to what extent rFE and rFD abolish each other during SSCs. Furthermore, the contribution of non-XB structures (as e.g., titin) to a potential SSC-effect in SSCs has to be examined.

This study aims to provide a systematic analysis of mechanical and contractile properties contributing to the increased force/work output in the shortening phase of SSCs compared to active shortening contractions without preceding stretch. To characterize the contribution of XB and non-XB structures to force/work production, we also performed experiments using the actomyosin inhibitor Blebbistatin. To achieve these goals, we performed *in vitro* isokinetic ramp experiments on single skinned skeletal muscle fibers obtained from the m. soleus of adult rats. Lengthening, shortening and SSC perturbations were conducted in the physiological range (along the ascending limb to the plateau region) of the force-length-relation and at fast contraction velocities of the soleus. The soleus muscle is an integral part of the triceps surae of the lower limb, operates as plantarflexor of the lower ankle joint and is mainly involved in SSCs during cyclic terrestrial locomotion of vertebrates.

## Materials and Methods

### Preparation, Handling and Experimental Set-Up

Muscle preparation, storage and activation techniques for permeabilized single muscle fibers were in line with Tomalka et al., 2017, 2019. Briefly, muscle fibers were extracted from seven freshly killed male Wistar rats (3–7 months, 425–500 g, cage-sedentary, 12 h:12 h light: dark cycle, housing-temperature: 22°C). The muscle fibers were obtained from soleus muscles from the left hind limbs. The Soleus is a predominantly slow-twitch skeletal muscle with a fiber type distribution of approximately 96% of type 1 fibers (Soukup et al., 2002). The skeletal muscle fibers from rats used for this study have been provided by another animal study that was approved according to the regulations of the German animal protection law (Tierschutzgesetz, §4 (3); Permit Number: 35-9185.81/0491). The applicants of the approved animal study had no objection against the extraction of muscle fibers from dead rats. The extraction of muscle fibers did not impair their results.

Fiber bundles were permeabilized at 4°C in a skinning solution (see section “Solutions”). Afterward, the demembranated fiber bundles were pinned at both ends — at approximately optimal *in vivo* sarcomere length — to a silicone elastomer surface. Subsequently, the fiber bundles were stored at −20°C in a storage solution (skinning solution made up in 50% glycerol) (see section “Solutions”) and used within 6 weeks. On the day of the experiments, small segments of the skinned fiber bundles were dissected under a stereomicroscope (Leica A60) and used to prepare several single muscle fibers (1.0–1.5 mm long) in a petri dish filled with storage solution positioned on a customized temperature-controlled stage at 4–6°C. After that, the fiber ends were loosely clamped by aluminum foil ‘T-clips’ (Institute of Applied Physics, Ultrafast Optics, Jena, Germany). Afterward, the fibers were treated with relaxing solution (see section “Solutions”) containing Triton X-100 (1% v/v) for 1–2 min at 4°C to ensure complete removal of internal membranes without affecting the contractile apparatus (Fryer et al., 1995; Linari et al., 2007). The fibers were either used immediately or stored overnight at −20°C in a storage solution. On the day of the experiments, the isolated muscle fiber was transferred to an experimental chamber (802D, Aurora Scientific, Canada) containing a relaxing solution. The clips of the fiber were attached to a force transducer (403a, Aurora Scientific, Canada) and a high-speed length controller (322 C-I, Aurora Scientific, Canada). Afterward, the fiber width (*w*) and height (*h*) were measured in approximately 0.1 mm intervals over the entire length with a 10× dry-objective (NA 0.30, Nikon) and a 10× eyepiece. The fiber cross-sectional area was determined assuming an elliptical cross-section of single muscle fibers (πhw/4) and was 4920 ± 1139 μm^2^. For visualization of the striation pattern and accurate, dynamic tracking of sarcomere length changes, a high-speed camera system (901B, Aurora Scientific, Canada) was used in combination with a 20 × ELWD dry-objective (NA 0.40, Nikon) and an accessory lens (2.5×, Nikon).

### Experimental Protocol

Each fiber was activated by calcium diffusion in the presence of ATP. The fiber was immersed in preactivating solution (see section “Solutions”) for 60 s for equilibration and afterward in an activating solution (pCa 4.5). This offered maximal activation that was characterized by a continuous rise in force until a plateau was reached (defined as a change in the force of less than 1% over a period of 5 s, achieved approx. 25 s after activation). Then, the ramp perturbations were applied to the fiber. Subsequently, fibers were immersed in relaxing solution for 420 s. Within this time interval of 420 s, a ‘cycling-protocol’ by Brenner (1983) was used to conserve the structural, functional and mechanical properties in maximally activated fibers over an extended period of time as well as to reduce sarcomere inhomogeneities. According to Tomalka et al. (2017), the following criteria were applied to discard fibers from the analysis: (1) isometric force in reference contractions was decreased by more than 10%; (2) abnormal behavior of force-traces, evidenced by artifacts, oscillations, or abrupt flattening was noted; and (3) lesions, ruptures or fiber contortion were identified visually.

**FIGURE 1:**
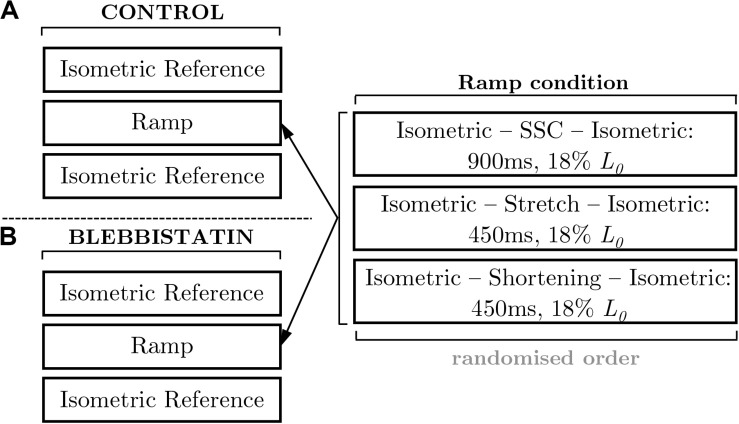
Schematic of the experimental protocol. The left panels illustrate the fixed protocol of muscle contractions consisting of isometric reference contractions and isokinetic ramp perturbations, for **(A)** control and **(B)** Blebbistatin experiments. The isometric reference measurements were performed in a randomized order at 0.82 *L*_0_ and 1.0 *L*_0_. The examination of varying ramp conditions followed a pseudorandomized block design (right panel). The applied stretch-shortening time periods correspond to a maximum shortening velocity (ν*_max_*) of 85% for all ramp conditions. The stretch-shortening magnitudes are normalized to the optimum muscle length (*L*_0_). SSC stands for stretch-shortening-cycle.

Isokinetic ramp perturbations comprised a set of repeated experiments. The control experiments (Figure 1) were designed to investigate the dynamic and static force response during and after isokinetic ramp perturbations. The Blebbistatin experiments are a repeat of the control experiments and involved the separation of the XB-contributions and non-XB-contributions to force production during and after isokinetic ramp perturbations (Figure 1).

**FIGURE 2:**
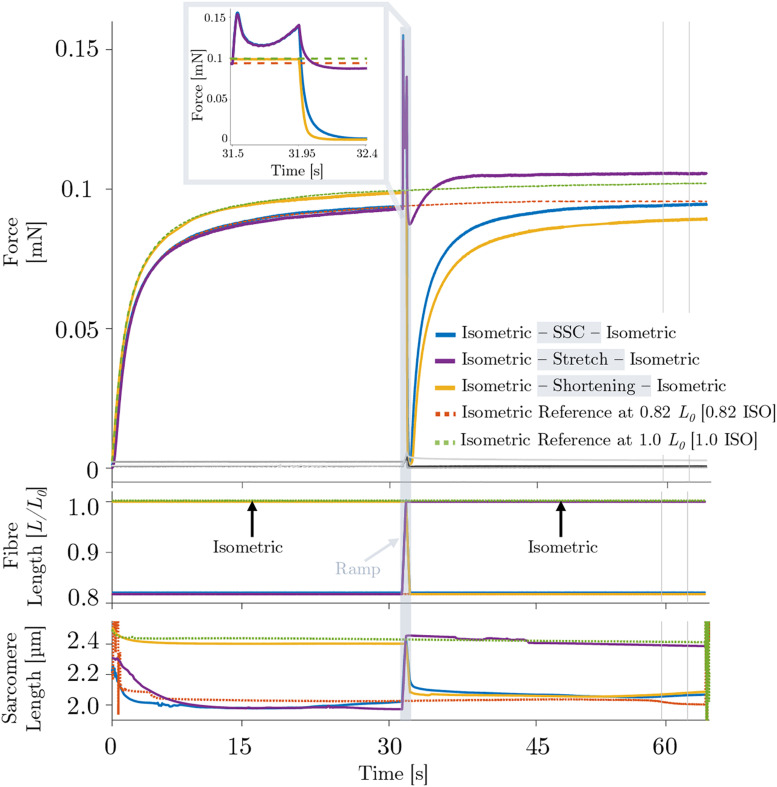
Representative force–time (upper graph), length–time (middle graph) and sarcomere length–time traces (lower graph) of a permeabilized single fiber segment from a rat soleus muscle (*n* = 1, raw, unfiltered data) at 12°C experimental temperature of the control experiment. The solid blue line indicates the stretch-shortening cycle (SSC), the solid purple line shows the stretch condition and the yellow solid line shows the shortening condition. The black line shows the passive SSC, the bright gray line the passive stretch and the dark gray line shows the passive shortening condition. Each of these experiments consists of an isometric phase, then a ramp transient, then an isometric phase. The red dashed line indicates the isometric reference contraction at 0.82 *L*_0_ (0.82 ISO), and the green dashed line shows the isometric reference contraction at optimum fiber length 1.0 *L*_0_ (1.0 ISO). After maximal isometric activation (pCa 4.5) until a plateau (defined as a change in the force of less than 1% for 5 s) was reached, isokinetic ramp perturbations have been applied to the tissue preparation (ramp initiation at *t* = 31.5 s). The force is shown in absolute values (mN). The fiber length is normalized to optimum muscle fiber length (*L*/*L*_0_). The sarcomere length is recorded at maximal activation and is shown in μm. All ramp experiments have been conducted at a constant stretch/shortening amplitude of 0.18 *L*/*L*_0_ within a constant amount of time (450 ms), respectively. The shaded rectangle indicates the period of the active stretch-shortening cycle (SSC) while the fiber segment is lengthened from 0.82*L*_0_ to 1.0*L*_0_ and immediately shortened to 0.82*L*_0_. The two vertical lines indicate the calculation period for rFE/rFD. Inset: enlarged view of the force response during an active SSC. For reasons of clarity, the passive traces have not been shown in the shaded rectangle or as part of the figure legend.

During the isokinetic ramp perturbations (control condition without XB-inhibition), single skinned muscle fibers (*n* = 16) were subjected to three different experiments, each consisting of an isometric phase, then a ramp transient, then an isometric phase (Figure 2). (*i*) For the SSC experiments (Figure 2, solid blue line), fibers were isometrically activated at 0.82 *L*_0_ (corresponding to ∼2.0 μm sarcomere length), stretched to optimum muscle length 1.0 *L*_0_ (∼2.5 μm sarcomere length) in 450 ms, and then immediately shortened to 0.82 *L*_0_ in 450 ms. (*ii*) For the active stretch trial (Figure 2, solid purple line), the fibers were isometrically activated at 0.82 *L*_0_ and lengthened to 1.0 *L*_0_ in 450 ms. (*iii*) For the active shortening trial (Figure 2, solid yellow line), fibers were isometrically activated at 1.0 *L*_0_ and then shortened to corresponding end length of 0.82 *L*_0_ in 450 ms. Due to consistency and simplification purposes, the following terms are used for the three different experiments: (*i*) SSC, (*ii*) stretch and (*iii*) shortening (Figure 2). For the investigation of individual isometric force responses following isokinetic ramp perturbations (rFE/rFD), the steady-state isometric contractions were sustained for 34.5 s at the final lengths (Figure 2, second half). To calculate rFE/rFD, we measured the average difference between the redeveloped and the corresponding isometric steady-state force at the same length — within a time interval of 5 s (28 s after the end of each ramp length change or cycle, cf. vertical lines of Figure 2).

The Blebbistatin experiments (*n* = 16) repeated the isokinetic ramps of the control experiments but in the presence of 20 μmol l^–1^ Blebbistatin in all solutions (see section “Solutions”). This photosensitive chemical is a selective inhibitor that blocks the force-generating transition of the bound actomyosin complex and causes myosin heads to bind to actin without exerting any force (Iwamoto, 2018). Blebbistatin does not affect titin mobility (Shalabi et al., 2017). The muscle fibers were treated with Blebbistatin in relaxation solution (pCa 9.0) for approximately 30 min in the dark. Throughout the experiments, the microscope room was maintained dark and a red-light filter (650 nm) was placed over the light source to prevent the breakdown of Blebbistatin when exposed to wavelengths between 365 and 490 nm (Kolega, 2004; Cornachione and Rassier, 2012).

All trials were performed at a constant velocity of 85% of the maximum shortening velocity (*v*_max_).

The *v*_max_ was defined as 0.48 *L_0_ s*^–^*^1^*, an average value (0.48 ± 0.13 *L_0_ s*^–^*^1^*; *n* = 4) of maximum unloaded shortening velocity of soleus muscle fibers from adult male Wistar rats. The individual *v*_max_-values were calculated based on own experimental data from isotonic contractions against forces in the range of 0.1 *F*_0_ to 0.9 *F*_0_. For the determination of force degradation, isometric reference contractions at *L*_0_ were performed before and after each ramp contraction. In ramp experiments (control), the isometric force in successive activations decreased at an average rate of around 3.3% per activation. The order of the ramp protocol was randomized. All experiments were conducted at a constant temperature of 12°C ± 0.1°C. At this temperature, the fibers proved very stable and able to withstand active ramp protocols over an extended period of time as well as prolonged activations (Ranatunga, 1982, 1984; Bottinelli et al., 1996; Tomalka et al., 2017).

### Solutions

The relaxing solution contained (in mM) 100 TES, 7.7 MgCl_2_, 5.44 Na_2_ATP, 25 EGTA, 19.11 Na_2_CP, 10 GLH (pCa 9.0). The preactivating solution contained (in mM) 100 TES, 6.93 MgCl_2_, 5.45 Na_2_ATP, 0.1 EGTA, 19.49 Na_2_CP, 10 GLH, 24.9 HDTA. The activating solution contained (in mM) 100 TES, 6.76 MgCl_2_, 5.46 Na_2_ATP, 19.49 Na_2_CP, 10 GLH, 25 CaEGTA (pCa 4.5). The skinning solution contained (in mM) 170 potassium propionate, 2.5 MgCl_2_, 2.5 Na_2_ATP, 5 EGTA, 10 IMID, 0.2 PMSF. The storage solution is the same as the skinning solution, except for the presence of 10 mM GLH and 50% glycerol (v/v). Cysteine and cysteine/serine protease inhibitors [*trans*-epoxysuccinyl-L-leucylamido-(4-guanidino) butane, E-64, 10 mM; leupeptin, 20 μg ml^–1^] were added to all solutions to preserve lattice proteins and thus sarcomere homogeneity (Linari et al., 2007; Tomalka et al., 2017). pH (adjusted with KOH) was 7.1 at 12°C. 450 U ml^–1^ of CK was added to all solutions, except for skinning and storage solutions. CK was obtained from Roche (Mannheim, Germany) and Blebbistatin was from Enzo Life Sciences Inc., NY, United States); all other chemicals from Sigma (St Louis, MO, United States).

### Data Processing and Statistics

Data were collected at 1 kHz. For data acquisition, real-time software (600A, Aurora Scientific, Canada) was used. For data analysis, a custom-written MATLAB (MathWorks, Natick, MA, United States) program was utilized. Unless stated otherwise, forces were expressed in absolute values (mN) and kilopascals (kPa) or normalized to the individual maximal muscle force (*F/F_0_*). The average active isometric force at optimum muscle length *L*_0_ was 0.31 ± 0.09 mN, this force corresponds to relative average stress values, normalized to the cross-sectional area, of 61.10 ± 10.93 kPa. Fiber lengths were expressed relative to the optimum fiber length (*L/L_0_*), while the mean *L*_0_ was 0.80 ± 0.10 mm. Sarcomere lengths were shown in absolute values (μm). Mechanical work was calculated as the line integral of the changing force over the entire shortening distance for both, the active shortening condition and the SSC condition are expressed in normalized values ($\int \frac{F}{F_{0}}\,\Delta \,\frac{L}{L_{0}}$). All data are presented as mean ± standard deviation (s.d.) unless stated otherwise. Parameters were tested for normal distribution using the Shapiro-Wilk Test. All data were normally distributed (*p* > 0.665). To test whether the steady-state isometric forces and sarcomere lengths differ between the different conditions ending at 0.82 *L*_0_ (SSC, shortening and isometric reference at 0.82 *L*_0_) an repeated-measures ANOVA was calculated. In case that the ANOVA demonstrated significant main effects, *post hoc* analyses were performed using the student’s *t*-test with Bonferroni correction. To determine significant differences in forces or sarcomere length when comparing the two conditions ending at 1.0 *L*_0_ (stretch and isometric reference at 1.0 *L*_0_), a student’s *t*-test was used. The statistical tests were likewise performed for both the control experiments and the Blebbistatin experiments. The level of significance was set at *p* < 0.05. Statistical analyses were realized using SPSS 25 (IBM Corp., Armonk, NY, United States). The effect sizes of Cohen’s *d* were calculated as $d=\frac{M_{1}-M_{2}}{S_{p\,o\,o\,l\,e\,d}}$, where *M* is the mean and *S*_*pooled*_ =$\sqrt{\frac{S\,D_{1}^{2}+S\,D_{2}^{2}}{2}}$ (Cohen, 1988). The effect sizes were classified as small (*d* = 0.2), medium (*d* = 0.5) and large (*d* = 0.8) (Cohen, 1988).

## Results

### Isometric Force Development After Isokinetic Ramp Contractions

**FIGURE 3:**
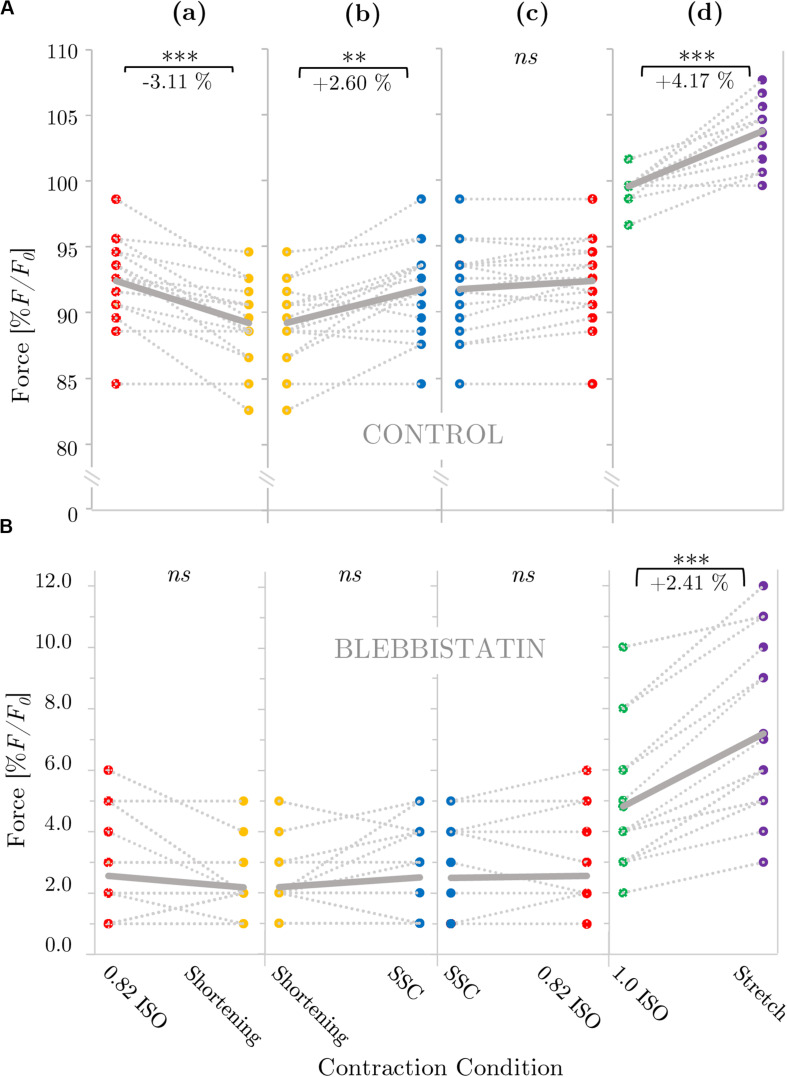
Influence of varying ramp experiments on force for **(A)** control and **(B)** Blebbistatin experiments. The gray dotted lines of the scatterplots shown in panels **(a–d)** indicate the individual paired data values and gray solid lines indicate the mean values (*n* = 16 fibers from seven rats). Two fibers per animal (median) were examined, except for one animal from which one fiber was examined and three animals from which three fibers were examined. Mean forces are normalized to the forces obtained during actively isometric reference contractions at corresponding end lengths (0.82 *L*_0_ [0.82 ISO; red circles] for active shortening [yellow circles] and stretch-shortening [SSC; blue circles] conditions, respectively, and 1.0 *L*_0_ [1.0 ISO; green circles] for active stretch [purple circles] conditions). **(B)** Statistical analyses are based on muscle fiber experiments in the presence of Blebbistatin. Forces are calculated 28–33 s after the end of each ramp and they are expressed in percentage of maximum isometric force (% *F/F_0_*). Brackets and asterisks (*) mark differences in force after varying ramp conditions in the intergroup comparison. Significance levels are marked as follows: ^∗^*p* < 0.05, ^∗^^∗^*p* < 0.01, and ^∗^^∗^^∗^*p* < 0.001. *ns* means not significant.

Figure 2 provides a representative overview of the forces produced by an isolated muscle fiber preparation during the different isokinetic ramps and the isometric conditions. For statistical comparison of the different contraction conditions, individual and mean isometric steady-state forces obtained 57.5–62.5 s after the start of each activation are shown in Figure 3A. Forces were significantly smaller (*p* ≤ 0.001, *d* = 0.98, Table 1) for the shortening condition [yellow circles, Figures 3A (a,b)] compared with the actively isometric reference contraction at corresponding end length of 0.82 *L*_0_ [0.82 ISO, red circles of Figures 3A (a,c)]. For the SSC condition [blue circles, Figures 3A (b,c)], isometric forces were not statistically different (*ns*) (*p* = 0.278, *d* = 0.15; Table 1) compared with 0.82 ISO. The comparison of active shortening and SSC revealed significantly larger (*p* = 0.002, *d* = 0.77) forces for the SSC condition [cf. Table 1 and Figure 3A (b)].

**TABLE 1:** Pairwise comparisons of isometric steady-state forces obtained during the control experiments.

**Control**						
**Δ*F/F_0_***	**Pairwise comparisons Δ *F/F_0_* [%]**				***n***	***p*-values**
	**Mean**	**s.d.**	**95% confidence interval**			
	**differences**		**of the difference**			
			**Lower**	**Upper**		
0.82 ISO - shortening	–3.11	2.56	0.77	5.44	16	<0.001
0.82 ISO - SSC	–0.51	1.31	–0.68	1.71	16	0.278
Shortening - SSC	2.60	2.61	–4.98	–0.21	16	0.002
1.0 ISO - stretch	4.17	2.17	–5.32	–3.00	16	<0.001

*Force values ± s.d. are normalized to the forces obtained during actively isometric reference contractions at corresponding end lengths (0.82 *L*_0_ [0.82 ISO] for active shortening and stretch-shortening [SSC] conditions, respectively and 1.0 *L*_0_ [1.0 ISO] for active stretch conditions). Forces are calculated 28–33 s after the end of each ramp and differences (Δ*F/F_0_*) are expressed in percentage (%). *ns* means not significant (*p* < 0.05). *n* is the number of samples.*

For the stretch condition [purple circles, Figure 3A (d)], mean isometric steady-state forces were significantly larger (*p* ≤ 0.001, *d* = 2.39; Table 1) compared with the corresponding isometric reference contraction at 1.0 *L*_0_ [1.0 ISO, green circles of Figure 3A (d)].

The corresponding sarcomere lengths for the SSC condition (2.02 ± 0.05 μm, blue line, Figure 2) were not statistically different (*ns*) (*p* = 0.238, *d* = 0.329) compared with 0.82 ISO (2.00 ± 0.07 μm, red dashed line, Figure 2) and compared with the shortening condition (2.03 ± 0.06 μm, *p* = 0.254, *d* = 0.181). For the stretch condition (2.39 ± 0.05 μm, purple line, Figure 2), the sarcomere lengths were not statistically different (*ns*) (*p* = 0.051, *d* = 0.340) compared with 1.0 ISO (2.38 ± 0.04 μm, green dashed line of Figure 2).

### Effects of Shortening and SSC on Mechanical Work

**FIGURE 4:**
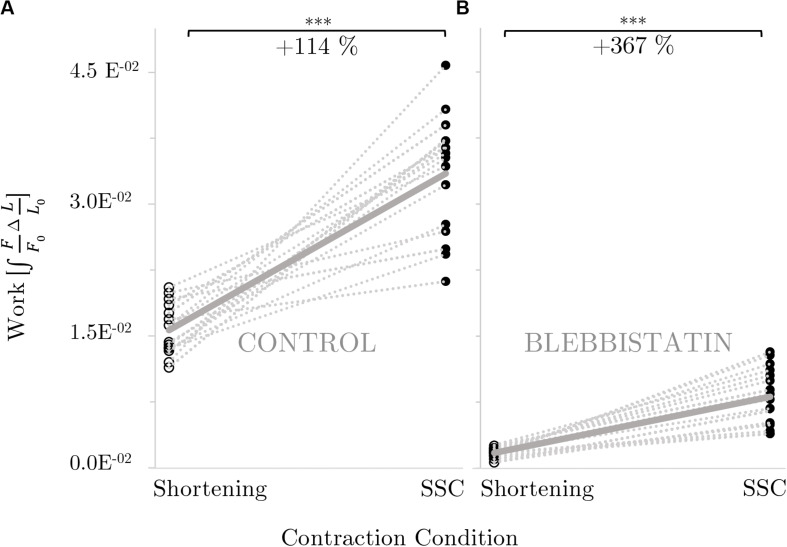
Influence of active shortening and SSC on work for **(A)** control and **(B)** Blebbistatin experiments. The gray dotted lines indicate the individual paired data values and the gray solid line indicates the mean value (*n* = 16). Work is shown in normalized values ($\int \frac{F}{F\,0}\Delta \frac{L}{L\,0})$. Brackets and asterisks (*) mark differences in work obtained during active shortening [white circles] compared with stretch-shortening [SSC; black circles] conditions in the intergroup comparison. **(B)** Statistical analysis is based on muscle fiber experiments in the presence of Blebbistatin. Significance levels are marked as follows: *p < 0.05, **p < 0.01, and ***p < 0.001. *ns* means not significant.

**FIGURE 5:**
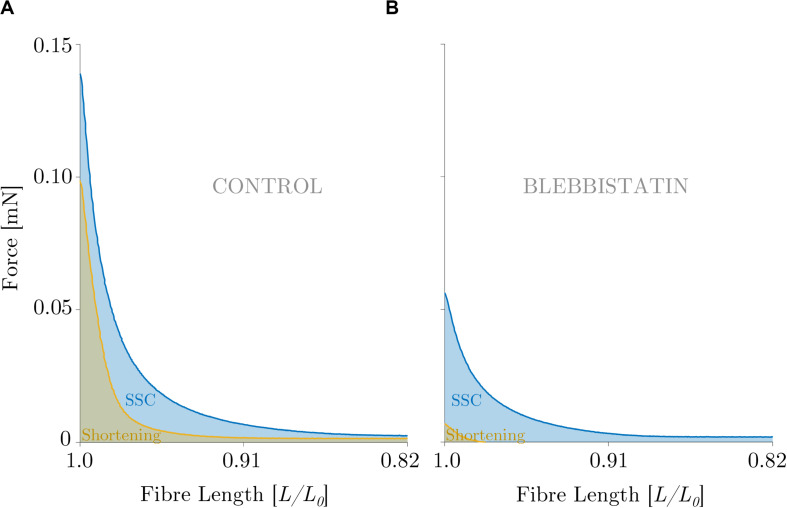
Direct visual comparison of mechanical work output obtained during the **(A)** control and the **(B)** Blebbistatin experiments. Representative force-length trace of a permeabilized single fiber segment from a rat soleus muscle (*n* = 1, raw data). Mechanical work was determined for a period of 450 ms during the shortening phase from the onset of release until the end of release using numerical integration of force with respect to length. The solid blue line indicates the shorting phase of the SSC, and the solid yellow line shows the active shortening condition. The blue and the brown shaded areas represent the line integrals for the SSC and shortening condition, respectively. The force is shown in mN and the fiber length is normalized to the optimum fiber length (*L/L_0_*). **(A)** The ‘CONTROL’ condition refers to the experiments without XB-inhibition, **(B)** ‘BLEBBISTATIN’ refers to the experiments with XB-inhibition.

Mechanical work was significantly larger for the SSC condition (black circles of Figure 4A) compared with the active shortening condition (white circles of Figure 4A) (*p* ≤ 0.001, *d* = 3.31). Figure 5A shows the distinct force responses and work (colored areas under the curves) during the shortening phase of the SSC condition and the shortening condition, respectively. While both conditions were subjected to the same amount of shortening (0.18 *L*_0_), mechanical work increased by 114% when shortening was preceded by a stretch (SSC condition) (Figure 4A).

### Effects of XB-Inhibition on Force Generation and Mechanical Work

#### Steady-State Isometric Force

**FIGURE 6:**
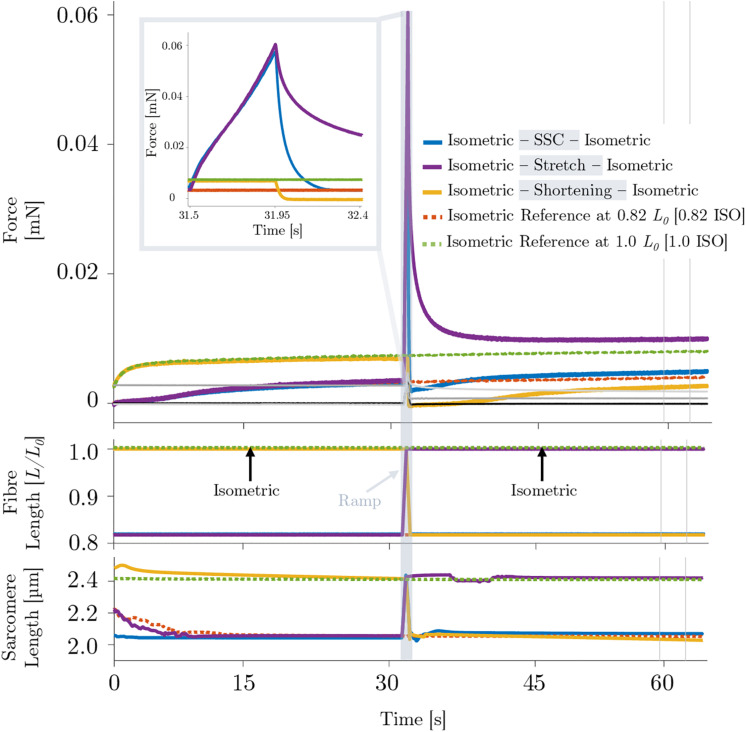
Representative force–time (upper graph), fiber length–time (middle graph) and sarcomere length–time traces of different isokinetic ramp perturbations (SSC, shortening, stretch) applied to a permeabilized single fiber segment from a rat soleus muscle (*n* = 1) of the Blebbistatin experiment. The actomyosin inhibitor Blebbistatin (20 μmol l^–1^) leads to marginal active isometric muscle force levels. Inset: enlarged view of the force response during an active SSC. For reasons of clarity, the passive traces have not been shown in the shaded rectangle or as part of the figure legend.

A representative example of the forces produced by an isolated muscle fiber preparation during the different isokinetic ramps and the isometric conditions in the presence of Blebbistatin is shown in Figure 6. Blebbistatin successfully inhibited active isometric muscle force and led to marginal levels of XB-based force production [about 5% *F*_0_ at optimal muscle length 1.0 *L*_0_; cf. Figure 3B (d)]. Mean isometric forces after the end of the isokinetic ramps revealed no significant differences for the active shortening condition [yellow circles of Figure 3B (a)] compared with 0.82 ISO [red circles of Figure 3B (a)] (*p* = 0.086, *d* = 0.44; cf. Table 2). For the SSC condition [blue circles of Figures 3B (b,c)], no statistically significant differences were found compared with 0.82 ISO (*p* = 0.686, *d* = 0.06; cf. Table 2) and compared with the shortening condition (*p* = 0.236, *d* = 0.38) [cf. Figures 3B (b,c)]. Comparison of 1.0 ISO and active stretch conditions [green vs. purple circles of Figure 3B (d)] revealed statistically greater forces (*p* ≤ 0.001, *d* = 0.91) for the stretch condition (cf. Table 2).

**TABLE 2:** Pairwise comparisons of isometric steady-state forces obtained during the Blebbistatin experiments.

**Blebbistatin**						
**Δ*F/F_0_***	**Pairwise comparisons Δ *F/F_0_* [%]**				***n***	***p*-values**
	**Mean**	**s.d.**	**95% confidence interval**			
	**differences**		**of the difference**			
			**Lower**	**Upper**		
0.82 ISO - shortening	–0.53	0.96	–0.35	1.41	16	0.086
0.82 ISO - SSC	–0.13	0.53	–0.36	0.62	16	0.686
Shortening - SSC	0.40	0.96	–1.28	0.48	16	0.236
1.0 ISO - stretch	2.41	1.12	–2.80	–1.82	16	<0.001

*Force values ± s.d. are normalized to the forces obtained during actively isometric reference contractions at corresponding end lengths (0.82 *L*_0_ [0.82 ISO] for active shortening and stretch-shortening [SSC] conditions, respectively, and 1.0 *L*_0_ [1.0 ISO] for active stretch conditions). Statistical analyses are based on experiments in the presence of Blebbistatin. Forces are calculated 28–33 s after the end of each ramp and differences (Δ*F/F_0_*) are expressed in percentage (%). ns means not significant (*p* < 0.05). *n* is the number of samples.*

The corresponding sarcomere lengths were not statistically different (*ns*) (*p* = 0.062, *d* = 0.392) for the SSC condition (2.13 ± 0.03 μm, blue line, Figure 6) compared with 0.82 ISO (2.12 ± 0.02 μm, red dashed line, Figure 6) and compared with the shortening condition (2.13 ± 0.04 μm, *p* = 0.654, *d* = 000). For the stretch condition (2.50 ± 0.02 μm, purple line, Figure 6), sarcomere lengths were not statistically different (*ns*) (*p* = 0.116, *d* = 0.632) compared with 1.0 ISO (2.49 ± 0.01 μm, green dashed line of Figure 6).

#### Effects of Shortening and SSC on Mechanical Work by Blebbistatin

Mechanical work was significantly higher for the SSC condition (black circles of Figure 4B) compared with the active shortening condition (white circles of Figure 4B) (*p* ≤ 0.001, *d* = 2.68). Figure 5B shows the distinct force responses and work (colored areas under the curves) during the shortening phase of the SSC condition and the shortening condition, respectively. Mechanical work increased by 367% for the SSC condition compared with shortening in the presence of Blebbistatin (cf. Figure 4B).

### Force-Kinetics During Isokinetic Ramp Perturbations

Comparing the active stretch during SSCs and stretch of the control condition without XB-inhibition (cf. Figure 2 inset, first half) with the active stretch during SSCs and stretch of the Blebbistatin condition with XB-inhibition (cf. Figure 6 inset, first half) revealed some distinct differences.

Without XB-inhibition, the muscle force increased steeply in the early phase of stretching. Then, up to about half of the stretching time, the force rapidly decreased with further stretching until it recovered by the end of the stretch (Figure 2 inset, first half). In the presence of Blebbistatin, the force response during the entire active stretch period is characterized by a progressive rise in force (Figure 6 inset, first half).

## Discussion

To the best of our knowledge, this study presents the first *in vitro* investigation of muscle fiber force/work production in physiologically relevant SSC conditions (a fast SSC along the ascending limb to the plateau region of the force-length-relation). To separate XB (contractile component) and non-XB (structural proteins such as titin) contributions to total muscle force in skinned muscle fibers, we used the actomyosin inhibitor Blebbistatin. Our experiments reveal three main results: (*i*) during the stretch phase of the SSC and stretch conditions, a substantial decline follows an initial steep increase in force until the force recovers more slowly — compared to the initial rise in force — up to the end of the stretch (Figure 2 inset, first half). Further, (*ii*) force output and work during shortening were significantly enhanced for SSCs compared with active shortening conditions (Figure 4A). (*iii*) No rFD was observed following SSCs compared with significant rFD following active shortening conditions [cf. blue vs. yellow line of Figures 2, 3A (b,c)].

### Isometric Forces Following Ramp Perturbations (Steady-State Phase)-Comparison to Other Studies

The steady-state isometric force following stretch was 4.2% higher (rFE), and the isometric steady-state force following active shortening was 3.1% smaller (rFD), relative to the corresponding isometric reference at the same fiber length (Table 1). Similar increases and decreases in force were observed in previous studies which used soleus fibers of the rat (Campbell and Moss, 2002), lumbrical muscles fibers of the frog (Bullimore et al., 2008), soleus muscles of the cat (Herzog and Leonard, 2000; Bullimore et al., 2007) as well as muscles from the hind limb of the rabbit (soleus, gastrocnemius, and plantaris) (Siebert et al., 2015).

There was no difference between the isometric reference force and the corresponding force in the steady-state phase following SSCs [Figure 3A (c)]. This result suggests that FE generated during the active lengthening phase of SSCs persisted during the subsequent shortening phase, thereby counteracting shortening-induced rFD when the shortening was preceded by stretching (SSC).

Also, the steady-state force after the SSCs was significantly higher by 2.6%, compared with the steady-state force after active shortening with the same magnitudes of shortening [Table 1 and Figure 3A (b)]. A similar increase in force was observed in a previous *in vitro* study using skinned soleus fibers of the rabbit (Fukutani and Herzog, 2019). Our results also agree with *in vivo* findings using muscles of the human plantar flexor and human adductor pollicis showing increased forces after SSCs compared with active shortening (Seiberl et al., 2015b; Hahn and Riedel, 2018).

### Influence of Contraction Velocity on XB-Dynamics During Stretch (Transient Phase)

Muscle fiber kinetics during the stretch phase of SSCs and active stretches were characterized by two consecutive peaks with a relatively compliant transient phase in between (cf. Figure 2 inset, first half). This distinct behavior has been referred to muscle ‘slippage’ or ‘give,’ in which the force redevelops more slowly after an internal ‘give’ (Katz, 1939; Flitney and Hirst, 1978; Griffiths et al., 1980; Choi and Widrick, 2010). It has been attributed to (partial) XB-detachment under strain when stretching velocities exceed a given threshold (Huxley, 1969; Sugi, 1972). This indicates that the peak force (referred to the first peak, cf. Figure 2 inset, first half) represents the force at which XBs are forcibly detached by the stretch (Bagni et al., 2005).

Under physiological conditions — e.g., during fast jumps or sprinting — the human soleus performs fast SSCs (<250 ms ground contact time) at moderate to high contraction velocities (at about 85% *v*_max_, equivalent to around 6 FL/s) (Ranatunga, 1984; Bobbert et al., 1986; Widrick et al., 1997; Komi, 2002; Fukashiro et al., 2006). By that, the human soleus’ operating range covers the ascending limb and the plateau-region of the force-length-relation (Burkholder and Lieber, 2001; Kurokawa et al., 2003). Our results indicate that at a high contraction velocity (as carried out in this study) XB-dynamics are altered compared with slow and moderate contraction velocities. This assumption is further based on an inherent muscle property which is attributed to short-range stiffness. Short-range stiffness is associated with a slightly damped stiffness with which active muscles resist small, rapid changes in length (Rack and Westbury, 1974; Campbell and Lakie, 1998). It is seen as the deformation of existing cross-bridges without compelling breakdown or reformation (Morgan, 1977). The initial steep linear rise in force upon active muscle stretching for extensions of 327–375 μm (equivalent to 1.16–1.34% *L*_0_) is followed by a negative force slope (Figure 2, inset). This transition phase resembles the ‘give’ termed S2 by Flitney and Hirst (1978) in frog muscle experiments. When the displacement of the filaments in the axial direction exceeds 11–12 nm, the XBs are forcibly detached and sarcomeres are no longer able to resist the rise in force upon active muscle stretching (Flitney and Hirst, 1978). Hence, only a fraction of attached XBs contributes to the total force response during the stretching phase of fast stretches and SSCs, while the other fraction of XBs becomes detached. Thus, short-range stiffness associated with ‘slippage’ – resulting in altered XB-dynamics – might have an impact on the magnitude of (r)FE (Fukutani et al., 2019).

Nevertheless, for stretch and SSCs starting from 0.82 *L*_0_, mean forces of 1.25 *F/F_0_* were observed at the end of the stretch where fibers were at the plateau region of the force-length-relation. These force magnitudes exceeded the maximum active forces produced by XBs at these lengths according to the sliding filament and XB-theories. It has been suggested that force enhancement may be caused by the engagement of titin during active muscle stretching (Rode et al., 2009; Edman, 2012; Tomalka et al., 2017; Herzog, 2018). Forces during the stretch exceeding *F*_0_ (Figure 2) are in line with findings by Tomalka et al. (2017) in skinned EDL muscle preparations. They observed maximum forces up to 2.5 *F/F_0_* on the descending limb of the force-length-relation during stretching with constant stretch amplitudes of 0.45 *L*_0_ at a given velocity of 10% *v*_max_. Comparable results can be expected for the soleus when tested under equal experimental conditions.

### Mechanical Work Output in SSCs vs. Active Shortening (Shortening Phase)

We examined the increase in mechanical work in SSCs compared with active shortening conditions.

The results of this study reveal greater work performed during shortening with preceding stretch (SSC) compared with shortening without preceding stretch (Figure 4A). Several factors may explain this increased work production, as they are: (*a*) increased elastic energy in the attached XBs, *(b*) contribution of active XB-forces, and (*c*) engagement of the giant filamentous structure titin.

When an activated muscle fiber is stretched, the attached XBs are also stretched and generate greater force (Huxley, 1957). It is assumed that elastic energy stored in elongated XBs, the myofilaments (Huxley et al., 1994; Wakabayashi et al., 1994) and crosslinking structures as the Z-disk (Luther, 2009; Burgoyne et al., 2015) may contribute to increased force and work during the stretch (Tomalka et al., 2017). As previously shown, peak forces at the end of an active stretch are higher for fast stretching velocities compared with slow stretching velocities (Edman et al., 1978; Sugi and Tsuchiya, 1988; Lombardi and Piazzesi, 1990). Consequently, the higher the stretching velocity, the higher the magnitude of elastic energy stored in XBs (Fukutani et al., 2017). Although fast stretching rates of 85% *v*_max_ (as carried out in this study) lead to, at least partial, detachment of bound XBs (‘slippage,’ cf. section “Influence of Contraction Velocity on XB-Dynamics During Stretch (Transient Phase)”), the reattachment of detached XBs is also very rapid (Lombardi and Piazzesi, 1990). Consequently, it can be speculated that elastic energy stored in XBs (due to the alteration of the XB-cycle) during stretching might contribute to the SSC-effect — albeit to a small fraction.

However, work in the shortening phase of the SSC condition was about twice as high than in the active shortening condition (Figures 4A, 5A). Consequently, factors other than altered XB-dynamics must have contributed to enhanced work output in SSCs.

Titin is known to be a significant contributor to the enhanced/depressed force response [(r)FE/(r)FD] during active stretch/shortening contractions (Rode et al., 2009; Leonard and Herzog, 2010; Joumaa et al., 2017; Dutta et al., 2018). Active muscle lengthening leads to increased steady-state isometric forces after stretch (rFE) (Figure 2). rFE is long-lasting and highly correlated with the force at the end of active muscle stretching (Noble, 1992; Bullimore et al., 2007). The findings of our study suggest that rFE generated during the active lengthening phase of SSCs persisted during the subsequent shortening phase, thereby contributing to the increased force output and work production (Seiberl et al., 2015b). Thus, we suggest that SSC-effects are likely related to rFE, which is in accordance to previous findings (Seiberl et al., 2015b; Fukutani et al., 2017; Fortuna et al., 2018; Hahn and Riedel, 2018; Fukutani and Herzog, 2019).

### Chemical XB-Inhibition by Blebbistatin

#### Isometric Forces Following Ramp Perturbations

The explanations for the SSC-effect given above are supported by the investigations of XB-contributions and non-XB-contributions to total muscle force (Blebbistatin condition) under different contraction conditions (SSC, shortening, stretch). The isometric steady-state force after stretch conditions was 2.4% higher, and the isometric steady-state force following shortening was not statistically different, relative to the corresponding isometric reference force at the same fiber length (Table 2). A similar increase in force (rFE) was observed in previous studies (using either Blebbistatin or BDM) on rabbit psoas muscle fibers (Cornachione and Rassier, 2012), mice soleus muscle fibers (Labeit et al., 2003) and frog tibialis anterior muscle (Bagni et al., 2002). A similar reduction, or even absence, of depressed forces (rFD) after shortening (during XB-inhibition) was observed in skinned psoas fibers from rabbits in the presence of BDM (Joumaa et al., 2012).

Assuming that potential titin-actin interactions do not require active force production and strong XB-binding to actin, the same contribution of titin-actin interactions to rFE would be expected in the Blebbistatin condition compared with the control condition. On the contrary, if titin-actin interactions depend on XB-force, no rFE is expected if Blebbistatin suppresses XB-force to a negligible level of maximum active force. The results of this study, obtained by administering Blebbistatin, revealed approximately 42% reduced rFE (relatively) due to 95% suppression of XB-interaction compared with the control condition (cf. results of Tables 1, 2). Thus, active XB-binding is necessary — at least partially — for the full development of rFE. These findings are in line with previous investigations (Leonard and Herzog, 2010; Powers et al., 2014) showing that XB-inhibition (using BDM) decreases the magnitude of (r)FE — compared with strong XB-binding. Furthermore, experiments on rabbit psoas myofibrils at very long lengths (>4 μm) (Leonard and Herzog, 2010) support this hypothesis, as no rFE was observed in the absence of actin-myosin overlap for myofibrils actively stretched from about 4.5 μm to 6 μm. Leonard and Herzog (2010) and Powers et al. (2014) suggested that either ‘active (actin-myosin based) force’ or XB-attachment to actin is required to produce rFE.

#### Influence of XB-Inhibition on Mechanical Work During Shortening

When the skinned soleus fibers were activated, then stretched, and immediately shortened (SSC condition), the work done during the shortening phase was about 3.7 times greater compared with the work during shortening (Blebbistatin condition) (Figure 4B). From our knowledge, this is the first study investigating mechanical work in muscle preparations with inhibition of XB-interaction.

The quasi-linear increase in force upon active muscle stretching (Figure 6 inset, first half), while actin–myosin interaction is suppressed, indicates that ‘slippage’ of attached XBs is dramatically reduced or even eliminated during fast active stretching in the presence of Blebbistatin. Therefore, the progressively increasing forces during active stretching at constant velocity indicate a continuous loading of non-XB elastic structures until the stretching has stopped. Consequently, stored elastic energy recoils in the shortening phase of SSCs. However, compared with the shortening phase of the SSC in the control condition (Figure 5A, the area below the blue line), work during the shortening phase of the SSC was reduced by 76% in the Blebbistatin condition (Figure 5B, the area below the blue line). This reduction in work is broadly in agreement with an observed decrease in rFE of 42% in the Blebbistatin compared with the control condition. Hence, reduced XB-binding by Blebbistatin (by 95%) might — at least partially — prevent titin-binding to actin and thus preloading of a shortened free titin spring during stretching.

There is an absolute work enhancement (work during the shortening phase of a SSC > work during active shortening) in both, the control and the Blebbistatin condition. However, this absolute work enhancement (Figure 5A, the area between the blue and the yellow line) was reduced by 64% in the Blebbistatin condition (Figure 5B, the area between the blue and the yellow line) compared to the control condition. Minor work enhancement under Blebbistatin might be, for example, due to the elastic energy that is stored and released in remaining XBs (approximately 5%) or a limited number of titin-actin interaction. Furthermore, calcium-induced stiffening of titin (Labeit et al., 2003; Joumaa et al., 2008b) might also contribute to minor work enhancement in SSCs compared with shortening, due to stretching of stiffer titin in SSC but not in active shortening.

Since Blebbistatin seems to affect the XB force, our approach does not clearly separate XB and non-XB contributions. Accordingly, alternative inhibitors as *N*-benzyl-p-toluene sulfonamide (BTS) should be considered for further studies attempting to separate XB and non-XB contributions (Iwamoto, 2018; Ma et al., 2018).

## Conclusion

The findings of this study reveal the following: (*I*) The SSC-effect is present at the single skinned muscle fiber level. Thus, there is direct evidence that the underlying mechanisms of the SSC-effect are within the sarcomere itself. (*II*) In the control condition, work and rFE are larger than in the Blebbistatin condition. From this, we conclude that XB-cycling contributes — at least partially — to the SSC-effect, which is likely to result from allowing titin-actin interaction. (*III*) The SSC-effect is still present in the Blebbistatin condition with a negligible number of active XBs. Consequently, non-XB structures contribute to the SSC-effect and rFE, probably through titin-actin interaction and calcium-induced stiffening of titin. To develop the full amount of increased titin-based force, active force production and strong XB-binding to actin is required. As this potentially titin-based increase in work is generated almost passively with no or negligible metabolic cost, based on our findings we conclude that titin contributes to the efficiency of SSCs. These assumptions are further supported by recent studies demonstrating that titin-based passive stress can activate the thick filament in skeletal muscle independent of calcium. This further supports a possible role of titin in the regulation of muscle contractility, likely mediated by the mechano-sensing signaling pathway in the myosin filament (Fusi et al., 2016; Brunello and Fusi, 2020).

The experimental findings of this study contribute to a detailed understanding of the SSC on the cellular level. With SSCs as the most basic everyday type of muscular contraction, this information not only promotes the basic understanding of muscle function underlying human locomotion but can also be used, i.e., for development of efficient humanoid drives with application in the field of movement science, medical engineering, robotics and prosthetics. Thus, there is a considerable significance of the implementation of SSC experiments in movement simulations — requiring a fundamental understanding of the underlying mechanisms.

## Data Availability Statement

All datasets presented in this study are included in the article/Supplementary Material.

## Ethics Statement

The studies involving animals were reviewed and approved according to the regulations of the German animal protection law (Tierschutzgesetz, §4 (3); Permit Number: 35-9185.81/0491) by the Regierungspräsidium Stuttgart, Department of Landwirtschaft, Ländlicher Raum, Veterinär- und Lebensmittelwesen.

## Author Contributions

TS, DH, WS, and AT contributed to the conceptualization of the study. SW and AT performed the experiments. AT analyzed the data and prepared the figures. AT, TS, DH, WS, and SW analyzed and discussed the results. AT and TS drafted the first version of the manuscript. AT, DH, TS, and WS edited and revised the manuscript. All authors contributed to the article and approved the submitted version.

## Conflict of Interest

The authors declare that the research was conducted in the absence of any commercial or financial relationships that could be construed as a potential conflict of interest.

## Abbreviations

ATP: adenosine 5 ′ triphosphate disodium salt hydrate
BDM: 2,3-butanedione monoxime
Ca^2+^: calcium
CK: creatine phosphokinase
CP: creatine phosphate
E-64: *trans*-epoxysuccinyl-L-leucylamido(4-guanidino)butane
EGTA: ethylene glycol-bis(2-aminoethylether)-*N*,*N*,*N*′,*N*′-tetraacetic acid
*F/F_0_*: maximum isometric muscle force
GLH: glutathione
HDTA: 1,6-diaminohexane-*N*,*N*,*N*′,*N*′-tetraacetic acid
*h*: height
IMID: imidazole
KOH: potassium hydroxide
KP: potassium propionate
*L/L_0_*: optimum muscle fiber length associated with *F/F_0_*
non-XB: non-cross-bridge
PMSF: phenylmethanesulfonyl fluoride
rFD: residual force depression
rFE: residual force enhancement
SSC: stretch-shortening cycle
TES: *N*-[tris(hydroxymethyl)methyl]-2-aminoethanesulfonic acid
v/v: volume/volume
*v*_max_: maximal shortening velocity
*w*: width
w/w: weight/volume
XB: cross-bridge.
