# Supplementary material for: Cross-Bridges and Sarcomeric Non-cross-bridge Structures Contribute to Increased Work in Stretch-Shortening Cycles
Source: Front Physiol. 2020 Jul 28;11:921. doi: 10.3389/fphys.2020.00921 (PMC7399218; doi:10.3389/fphys.2020.00921)
Supplement: Supplementary file 1 [file Data_Sheet_1.docx]

**Appendix**

**Cross-bridges and sarcomeric non-cross-bridge structures contribute to increased work in stretch-shortening cycles**

**André Tomalka^1*^, Sven Weidner^1^, Daniel Hahn^2,3^, Wolfgang Seiberl^4^, Tobias Siebert^1^**

^1^Department of Motion and Exercise Science, University of Stuttgart, Stuttgart, Germany

^2^Human Movement Science, Faculty of Sports Science, Ruhr University Bochum, Bochum, Germany

^3^School of Human Movement and Nutrition Sciences, University of Queensland, Brisbane, Australia

^4^Human Movement Science, Bundeswehr University Munich, Munich, Germany

***Correspondence:**

André Tomalka
andre.tomalka@inspo.uni-stuttgart.de

**Supplementary text S1**

**Potential mechanisms of history-effects**

FE in skeletal muscle during and following active stretches is associated with increased performance at decreased oxygen consumption, reduced metabolic cost (ATP), improved energy efficiency, as well as decreased muscle activity (Cavagna et al., 1968; Joumaa and Herzog, 2013; Nishikawaa et al., 2018; Powers et al., 2016; Seiberl et al., 2015a). FE exists during voluntary contractions and is relevant for movement generation in daily activities (Seiberl et al., 2015a). FE is in the meantime a generally accepted property and represents an important determinant of active force production (Campbell and Campbell, 2011; Herzog et al., 2016; Rassier, 2017; Rode et al., 2009; Siebert et al., 2015). Moreover, the linear spring behaviour in skeletal muscle, might offer high impact shock absorption strategies during eccentric movements such as downhill running or landing after jumps (Tomalka et al., 2017).

Several model approaches have been proposed that explain history-effects ((r)FE & (r)FD) in skeletal muscle based on an adjustable titin spring (Heidlauf et al., 2017; Nishikawa et al., 2012; Rode et al., 2009). These approaches are backed by a large number of experimental evidence for titin-actin interactions upon muscle activation (Astier et al., 1998; Bianco et al., 2007; Dutta et al., 2018; Li et al., 2018; Nagy, 2004; Tahir et al., 2020). For recent reviews on the mechanisms of (r)FE see (Herzog et al., 2016; Rassier, 2017).

One of the key mechanisms suggested for (r)FD is the stress-induced inhibition of XBs in the actin-myosin overlap zone (Herzog, 2004; Joumaa et al., 2017; Maréchal and Plaghki, 1979). The decrease in the number of cycling XBs has been associated with the amount of mechanical work performed during the shortening phase (Herzog et al., 2000; Seiberl et al., 2015b). A second main mechanism for (r)FD is attributed to titin. *Rode et al.* (2009) suggest that titin binds to actin upon muscle activation, which leads to a reduction in the persistence length of titin. This, in turn, is associated with an inhibition of XBs (less binding sites for myosin on the actin filament due to bound titin), which leads to a reduced or even negative passive & active force during shortening contractions.

**Supplementary Figure F1**

Isometric – SSC – Isometric

Isometric Reference at 0.82 *L_0_* [0.82 ISO]

Isometric Reference at 1.0 *L_0_* [1.0 ISO]

Isometric – Shortening – Isometric

Isometric – Stretch – Isometric

Figure F1.Influence of varying ramp experiments on force for control (A) and Blebbistatin (B) conditions. Representative total force-time (upper graphs) and corresponding passive force-time traces (lower graphs) of a permeabilised single fibre segment from a rat soleus muscle (n = 1, raw, unfiltered data) at 12°C experimental temperature. The solid blue line indicates the stretch-shortening cycle (SSC), the solid purple line shows the stretch condition and the yellow solid line shows the shortening condition. The red dashed line indicates the isometric reference contraction at 0.82 L_0_ (0.82 ISO), and the green dashed line shows the isometric reference contraction at optimum fibre length 1.0 L_0_ (1.0 ISO).

B

A


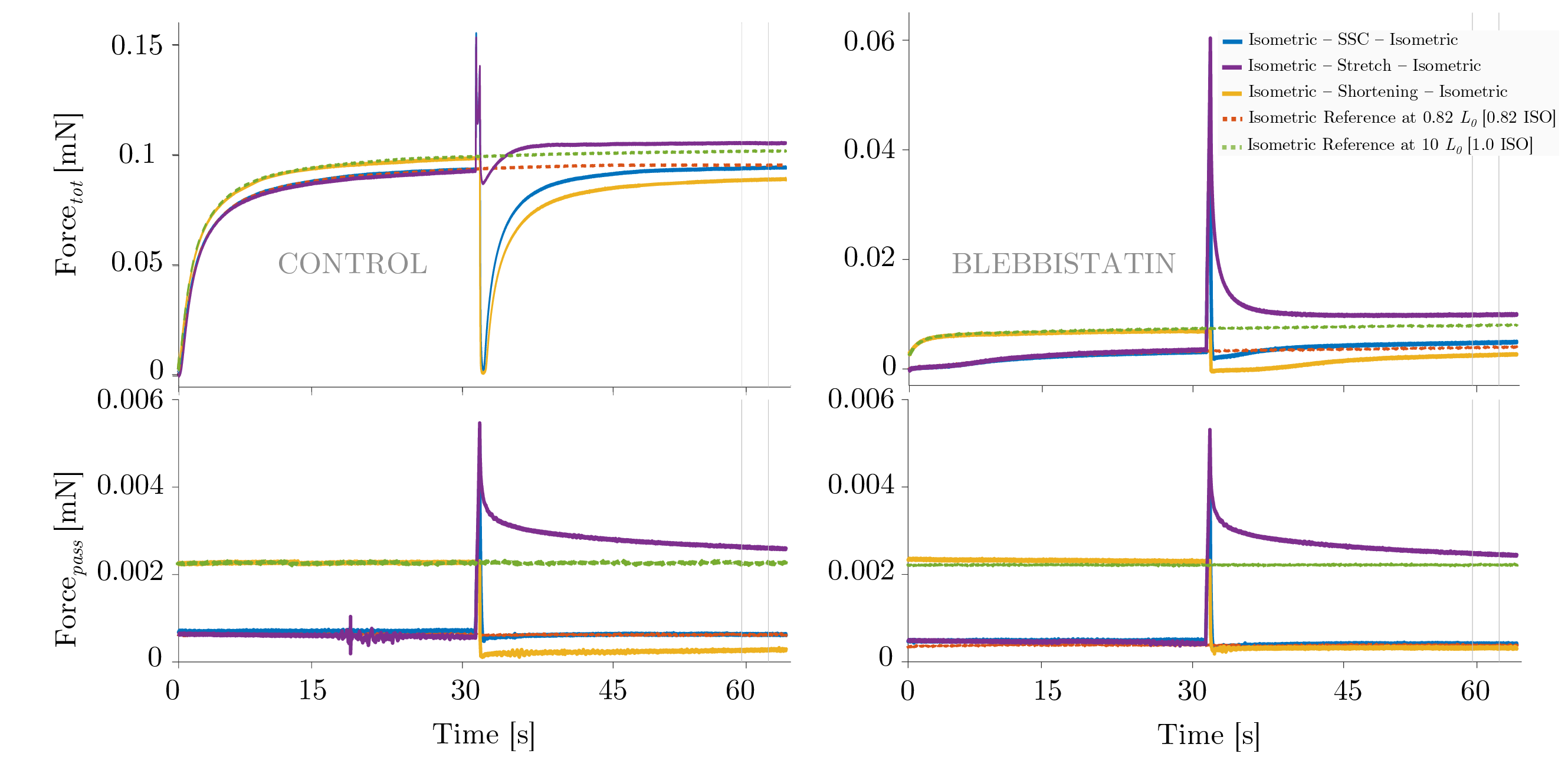


**References**

Astier, C., Raynaud, F., Lebart, M. C., Roustan, C., and Benyamin, Y. (1998). Binding of a native titin fragment to actin is regulated by PIP2. *FEBS Lett.* 429, 95–98. doi:10.1016/S0014-5793(98)00572-9.

Bianco, P., Nagy, A., Kengyel, A., Szatmári, D., Mártonfalvi, Z., Huber, T., et al. (2007). Interaction forces between F-actin and titin PEVK domain measured with optical tweezers. *Biophys. J.* 93, 2102–2109. doi:10.1529/biophysj.107.106153.

Campbell, S. G., and Campbell, K. S. (2011). Mechanisms of residual force enhancement in skeletal muscle: Insights from experiments and mathematical models. *Biophys. Rev.* 3, 199–207. doi:10.1007/s12551-011-0059-2.

Cavagna, G. A., Dusman, B., and Margaria, R. (1968). Positive work done by a previously stretched muscle. *J. Appl. Physiol.* 24, 21–32.

Dutta, S., Tsiros, C., Sundar, S. L., Athar, H., Moore, J., Nelson, B., et al. (2018). Calcium increases titin N2A binding to F-actin and regulated thin filaments. *Sci. Rep.* 8, 1–11. doi:10.1038/s41598-018-32952-8.

Heidlauf, T., Klotz, T., Rode, C., Siebert, T., and Röhrle, O. (2017). A continuum-mechanical skeletal muscle model including actin-titin interaction predicts stable contractions on the descending limb of the force-length relation. *PLoS Comput Biol.* 13, 1–25.

Herzog, W. (2004). History dependence of skeletal muscle force production: implications for movement control. *Hum. Mov. Sci.* 23, 591–604. doi:10.1016/j.humov.2004.10.003.

Herzog, W., Leonard, T. R., and Wu, J. Z. (2000). The relationship between force depression following shortening and mechanical work in skeletal muscle. *J. Biomech.* 33, 659–68. Available at: http://www.ncbi.nlm.nih.gov/pubmed/10807986.

Herzog, W., Schappacher, G., DuVall, M., Leonard, T. R., and Herzog, J. A. (2016). Residual Force Enhancement Following Eccentric Contractions: A New Mechanism Involving Titin. *Physiology* 31, 300–312. doi:10.1152/physiol.00049.2014.

Joumaa, V., Fitzowich, A., and Herzog, W. (2017). Energy cost of isometric force production after active shortening in skinned muscle fibres. *J. Exp. Biol.* 220, 1509–1515. doi:10.1242/jeb.117622.

Joumaa, V., and Herzog, W. (2013). Energy cost of force production is reduced after active stretch in skinned muscle fibres. *J. Biomech.* 46, 1135–9. doi:10.1016/j.jbiomech.2013.01.008.

Li, Y., Unger, A., von Frieling-Salewsky, M., Rivas Pardo, J. A., Fernandez, J. M., and Linke, W. A. (2018). Quantifying the Titin Contribution to Muscle Force Generation using a Novel Method to Specifically Cleave the Titin Springs in Situ. *Biophys. J.* 114, 645a. doi:10.1016/j.bpj.2017.11.3480.

Maréchal, G., and Plaghki, L. (1979). The deficit of the isometric tetanic tension redeveloped after a release of frog muscle at a constant velocity. *J. Gen. Physiol.* 73, 453–67. doi:10.1085/jgp.73.4.453.

Nagy, A. (2004). Differential actin binding along the PEVK domain of skeletal muscle titin. *J. Cell Sci.* 117, 5781–5789. doi:10.1242/jcs.01501.

Nishikawa, K. C., Monroy, J. A., Uyeno, T. E., Yeo, S. H., Pai, D. K., and Lindstedt, S. L. (2012). Is titin a “winding filament”? A new twist on muscle contraction. *Proc. R. Soc. B Biol. Sci.* 279, 981–990. doi:10.1098/rspb.2011.1304.

Nishikawaa, K., Lindstedt, S., and LaStayo, P. (2018). Basic science and clinical use of eccentric contractions: history and uncertainties. *J. Sport Heal. Sci.* in press. doi:10.1016/j.jshs.2018.06.002.

Powers, K., Nishikawa, K., Joumaa, V., and Herzog, W. (2016). Decreased force enhancement in skeletal muscle sarcomeres with a deletion in titin. *J. Exp. Biol.* 219, 1311–1316. doi:10.1242/jeb.132027.

Rassier, D. E. (2017). Sarcomere mechanics in striated muscles: from molecules to sarcomeres to cells. *Am. J. Physiol. - Cell Physiol.* 313, C134–C145. doi:10.1152/ajpcell.00050.2017.

Rode, C., Siebert, T., and Blickhan, R. (2009). Titin-induced force enhancement and force depression: a “sticky-spring” mechanism in muscle contractions? *J. Theor. Biol.* 259, 350–360. doi:10.1016/j.jtbi.2009.03.015.

Seiberl, W., Power, G. A., and Hahn, D. (2015a). Residual force enhancement in humans: Current evidence and unresolved issues. *J. Electromyogr. Kinesiol.* 25, 571–580. doi:10.1016/j.jelekin.2015.04.011.

Seiberl, W., Power, G. a., Herzog, W., and Hahn, D. (2015b). The stretch-shortening cycle (SSC) revisited: residual force enhancement contributes to increased performance during fast SSCs of human m. adductor pollicis. *Physiol. Rep.* 3, e12401–e12401. doi:10.14814/phy2.12401.

Siebert, T., Leichsenring, K., Rode, C., Wick, C., Stutzig, N., Schubert, H., et al. (2015). Three-Dimensional Muscle Architecture and Comprehensive Dynamic Properties of Rabbit Gastrocnemius, Plantaris and Soleus: Input for Simulation Studies. *PLoS One* 10, e0130985. doi:10.1371/journal.pone.0130985.

Tahir, U., Monroy, J. A., Rice, N. A., and Nishikawa, K. C. (2020). Effects of a titin mutation on force enhancement and force depression in mouse soleus muscles. *J. Exp. Biol.* 223. doi:10.1242/jeb.197038.

Tomalka, A., Rode, C., Schumacher, J., and Siebert, T. (2017). The active force – length relationship is invisible during extensive eccentric contractions in skinned skeletal muscle fibres. *Proc. R. Soc. B Biol. Sci.* 284, 20162497. doi:10.1098/rspb.2016.2497.
